# Supplementary material for: In silico evaluation of garlic-derived organosulfur compounds as multi-target inhibitors of breast cancer biomarkers
Source: PLoS One. 2026 May 6;21(5):e0348369. doi: 10.1371/journal.pone.0348369 (PMC13148666; doi:10.1371/journal.pone.0348369)
Supplement: S1 File — (DOCX) [file pone.0348369.s001.docx]

**SUPPLEMENTAL DATA**

| **Target Protein** | **Center Coordinates (x, y, z)** | **Grid Size (Å³)** | **Grid Spacing (Å)** |
| --- | --- | --- | --- |
| Bcl-2 | -0.024, 3.142, -0.361 | 65 × 65 × 65 | 0.375 |
| CDK6 | 2.296, 36.095, 138.519 | 60 × 60 × 60 | 0.375 |
| CDK2 | -7.623, 49.881, 11.367 | 60 × 60 × 60 | 0.375 |
| VEGFR2 | 5.396, 32.493, 15.884 | 70 × 70 × 70 | 0.375 |
| XIAP-Bir2 | 29.799, 34.241, 11.358 | 50 × 50 × 50 | 0.375 |
| G-quadruplex | 15.456, 16.903, 7.206 | 45 × 45 × 60 | 0.375 |
| Topoisomerase I | 21.171, -3.904, 25.952 | 70 × 70 × 70 | 0.375 |
| Topoisomerase II | 39.262, -1.072, 37.077 | 60 × 60 × 60 | 0.375 |

Table S1. Grid box parameters used for molecular docking of breast cancer targets.
