# Supplementary material for: In silico evaluation of garlic-derived organosulfur compounds as multi-target inhibitors of breast cancer biomarkers
Source: PLoS One. 2026 May 6;21(5):e0348369. doi: 10.1371/journal.pone.0348369 (PMC13148666; doi:10.1371/journal.pone.0348369)
Supplement: Attachment — Submitted filename: Rebutal letter.docx [file pone.0348369.s003.docx]

Department of Clinical Pathology

Noguchi Memorial Institute for Medical Research,

College of Health Sciences, University of Ghana

P. O. Box LG 581, Legon, Accra, Ghana.

September 20, 2025

Dear Dr. Al-Karmalawy,

We appreciate the time and effort you and the reviewers have dedicated to the thorough examination of our manuscript titled ‘In silico evaluation of garlic-derived organosulfur compounds as multi-target inhibitors of breast cancer biomarkers.’ Your constructive feedback has been invaluable in improving the overall quality and clarity of our work. To address your comments, we have carefully reviewed and revised the manuscript to address all concerns raised by the reviewers.

We believe that the manuscript is now suitable for publication in PLOS ONE.

Sincerely,

Kwabena O. Danquah

Corresponding author (on behalf of all authors)

In each sample, the review comments are not italicized while the responses are italicized.

**Reviewer #1**

**Comment 1**

Include molecular dynamics (MD) simulations or post-docking energy minimization to assess the stability of protein-ligand complexes over time. This is crucial to support docking results

**Response**

*Thank you for your valuable feedback and for suggesting the inclusion of molecular dynamics simulations or post-docking energy minimization to evaluate the stability of protein-ligand complexes over time. In response, we have performed post-docking energy minimization to assess the stability of the protein-ligand complexes. Details of this additional analysis have been incorporated into the Methodology (lines 134-146), and the corresponding findings are presented in the Results (lines 251-263) and illustrated in Figure 5. Furthermore, these results have been discussed in the Discussion section to reflect their implications*.

**Comment 2**

Because of the weak binding affinity the author need to discuss the therapeutic relevance of such moderate affinities in more detail, and consider whether structure-based optimization could improve potency.

**Response**

*We thank the reviewer for this valuable suggestion. In the original version of the manuscript, we had included a note on the potential for structural optimization to improve the potency of the compounds. In the revised manuscript, we have expanded this discussion to also address the therapeutic relevance of the weak binding affinities observed. This has now been incorporated into the Discussion section (lines 411-414). We believe that this addition provides a more balanced perspective on the translational potential of the compounds and highlights possible avenues for future drug development*.

**Comment 3**

Inadequate Comparison with Existing Drugs, author need to provide comparative data or references to contextualize how garlic compounds perform relative to standard breast cancer drugs.

**Response**

*We thank the reviewer for this important observation and suggestion. In the revised Discussion, we have expanded our comparisons between garlic-derived compounds and FDA-approved inhibitors as well as previously reported in silico studies. Specifically, we now compare S-allyl-L-cysteine’s binding to CDK6 with that of Abemaciclib, an FDA-approved CDK4/6 inhibitor (ΔG ≈ -12.12 kcal/mol vs -5 kcal/mol, respectively), and highlight how S-allyl-L-cysteine engages critical residues in the ATP-binding pocket while offering potential advantages in biocompatibility and reduced toxicity. We also discuss how rational structural modifications could enhance its affinity and selectivity, drawing from published interaction patterns of Abemaciclib. Similarly, Z-ajoene’s binding to CDK-2 and topoisomerase II is contextualized by comparison with palbociclib and etoposide, respectively. These additions provide a clearer framework for interpreting our results relative to clinically relevant standards. The new text can be found in the Discussion section, lines 388-399.*

**Comment 4**

Conclusion emphasizes therapeutic potential despite no experimental (in vitro/in vivo) validation. Author needs to acknowledge that findings are preliminary and hypothesis-generating, pending experimental confirmation.

**Response**

*We thank the reviewer for this valuable feedback. While we had noted in the original submission that the findings are preliminary and require experimental validation, we agree that this point needed to be emphasized more strongly. In the revised manuscript, we have made this limitation explicitly clear in the Abstract and Conclusion, highlighting that our results should be regarded as hypothesis-generating and that further in vitro and in vivo validation will be essential to confirm the therapeutic potential of the identified compounds against breast cancer targets. We believe this addition provides a more balanced and transparent interpretation of the study outcomes.*

**Comment 5**

The manuscript lacks a clear justification for why the 8 specific targets (e.g., CDK2, VEGFR2, G-quadruplex) were chosen. Author needs to justify the clinical relevance of each target in breast cancer pathophysiology and resistance mechanisms

**Response**

*We thank the reviewer for this valuable feedback. While the original manuscript (lines 54–64) provided a general rationale for selecting the eight targets, we agree that a clearer justification was needed. In the revised version, we have expanded the Background section (lines 64-66) to emphasize that these macromolecules are currently targeted by existing breast cancer therapies and to highlight the clinical relevance of each target in breast cancer pathophysiology and resistance. For example, CDKs and topoisomerases are linked to unchecked proliferation and endocrine resistance, Bcl-2 and XIAP mediate apoptosis evasion and chemoresistance, VEGFR2 is central to angiogenesis and tumor progression, and G-quadruplex structures play a role in telomerase regulation and genomic instability. This addition provides an additional layer of information to support the selection of these specific targets and shows their therapeutic importance in drug-resistant breast cancer.*

**Comment 6**

Figures lack resolution, and tables are dense without highlighting top-performing compounds.

**Response**

*We thank the reviewer for this important observation. In response, we have revised the relevant figures and tables to improve clarity and readability. Specifically, in Table 1, the top-performing docked compounds against each macromolecule have been highlighted in bold and a table legend added for easy identification. In addition, all figures have been updated with high-resolution images (≥300 dpi) to ensure better quality in the revised manuscript.*

**Comment 7**

Certain points (e.g., garlic's anticancer potential) are repeated verbatim. Condense overlapping information to improve focus and flow.

**Response**

*We thank the reviewer for this observation. In the revised manuscript, we have carefully reviewed and condensed sections where overlapping information was presented, particularly regarding garlic’s anticancer potential. Redundant statements have been removed or rephrased to improve focus, clarity, and flow, ensuring that each point is presented only once in the most appropriate context.*

**Reviewer #2**

**Comment**

In the molecular modelling simulation section, brief description regarding the topology and binding site description of the five investigated breast cancer-associated biotarget’s. Further, the key reported binding residues should be highlighted as being mentioned important within the current literature. Further, the compounds’ polar interaction patterns with key pocket residues should be annotated in terms of both the bond distances and angles. Hydrogen bonding should be presented within hydrogen bond distances as well as bond angles since hydrogen bond depend on both. Authors should mention the Hydrogen bond angles as well as their distances, since the strength of hydrogen bonding is based on both parameters in a way to ensure the adequacy of optimum hydrogen bonding.

**Response**

*Thank you for the feedback. In response to your suggestion, we have annotated the bond angles, distances, and key binding residues in Figure 4 and discussed them in the results section. Additionally, our study includes eight breast cancer-associated targets, not five. For the binding site descriptions, we have referenced the databases from which the targets were retrieved, as these sources provide comprehensive details on their topology and binding sites. We believe repeating this information in our manuscript would be redundant. However, we have included the resolution and PDB IDs of the targets around lines 118-121. In our methodology, we explained that the binding sites were selected based on known inhibitors, with their coordinates provided. Our analysis focuses on the residues in these regions that interact with our ligands (compounds), identifying key interacting residues within that known binding site to guide the further development of the lead compounds identified in our study.*

**Comment**

The authors should provide MM_GBSA or PBSA energy calculations. It is advised to provide the dissected energy terms of these total energies (ΔGbind Lipo, ΔGbind Solv GB, ΔGbind vDW, ΔGbind Coulomb, and ΔGbind Ligand strain) to further evaluate the nature of interaction (dominant energy potential) based on these different energy terms. This would guide further hit to lead and lead optimization steps.

**Response**

*Thank you for your suggestion and this important observation. Based on suggestions by other reviewers, we proceeded to do post-docking energy minimization to evaluate the stability of the protein-compound complexes. For individual residues contributing to energy, we have annotated residues that the ligands (compounds) interacted with and the type of bonds they formed. MM_GBSA or PBSA energy calculations would be valuable after MD simulation in our opinion but as said earlier, based on recommendations, we went ahead to do post-docking energy minimization which we have described at appropriate sections in the revised manuscript.*

**Comment**

Based on the study results, what are the take-away messages. Authors are advised to highlight the future suggested structural modifications that would improve the hits’ predicted activities based on the computational findings. These insights would be beneficial for guiding future lead optimization and development.

**Response**

*Thank you for your suggestion. We have made suggestions for the kind of approach that can be used for the structural optimization around lines 396-399. The key take-away messages have also been highlighted in the conclusion of the manuscript.*
