# Supplementary material for: In silico evaluation of garlic-derived organosulfur compounds as multi-target inhibitors of breast cancer biomarkers
Source: PLoS One. 2026 May 6;21(5):e0348369. doi: 10.1371/journal.pone.0348369 (PMC13148666; doi:10.1371/journal.pone.0348369)
Supplement: Attachment — Submitted filename: Response to the Reviewers.docx [file pone.0348369.s005.docx]

To the Editor,

**RE: RESPONSE TO THE REVIEWERS**

In each sample, the review comments are not italicized while the responses are italicized.

**Reviewer 2**

No responses received

**Reviewer 3**

**Comment**

1. In the Introduction section, the authors should include a brief overview of recent in silico techniques, supported by relevant references, to align with the title and provide better context for the study. This will enhance the reader’s understanding of the computational approaches used.

**Response**

*Thank you for this important feedback. In the revised version of the manuscript, we have now provided a clearer rationale for the use of in silico approaches in cancer drug discovery and included a brief overview of key computational techniques, supported by relevant references. A notable example added is the use of in silico techniques to discover a novel inhibitor against HER2 and its subsequent experimental validation which proved its micropotency. This information has been incorporated into the Introduction to better align with the study’s focus and to help readers understand the significance and context of the computational methods employed. This can be found on lines 42-55.*

**Comment**

2. Docking requires 3D coordinates. Please state explicitly how the 2D PubChem records were converted to 3D (e.g., OpenBabel, RDKit, Avogadro, Chem3D, or a PubChem 3D conformer download) and include the software name and version.

3. Describe how protonation states (pH considered), stereochemistry, and tautomeric forms were handled before optimization and docking. Indicate whether explicit hydrogens were added and which protonation state was used.

**Response (to comments 2 and 3)**

*Thank you for your valuable feedback regarding our ligand preparation methodology. For the file formats and conversions needed for the molecular docking, we have updated the manuscript around lines 114 to 124 to clearly communicate it. We confirmed that all ligands were processed to ensure the correct major microspecies at physiological pH (7.4) using the OpenBabel pH flag (pH 7.4) during the SDF to MOL2 conversion. For stereochemistry, the (S) absolute configuration was chosen for cpd6, as this is the only chiral ligand and this stereoisomer is experimentally reported as potent, and for compound cpd1, the (Z) geometric isomer was maintained because it was experimentally shown to be potent in relevant studies. Finally, we employed ACD/ChemSketch to validate that the MOL2 files represented the most thermodynamically favorable tautomer. The structures incorporating these finalized definitions (protonation, stereochemistry, and tautomerism) are all depicted in Figure 1 of the revised manuscript around lines 121 to 123.*

**Comment**

4. Although MMFF94 and steepest descent are mentioned, the manuscript should specify the program or server used to perform the optimization (including version), the implementation of MMFF94, the maximum number of optimization steps or exact stopping criterion (rather than “varied until energy minimization was achieved”), and the final energy or RMS gradient reached.

**Response**

*Thank you for the comment. We now specify that ligand optimization was performed in OpenBabel (version 2.4.1) using MMFF94 with steepest descent for a maximum of 600 steps. We have updated our original statement of ‘ Geometry optimization was carried out using the MMFF94 force field and steepest descent (maximum 600 steps) algorithm with a convergence criterion of 1 × 10⁻⁷’ to ‘Geometry optimization was carried out using the MMFF94 force field and steepest descent (maximum 600 steps) algorithm with an RMS gradient convergence criterion of 1 × 10⁻⁷ after converting the SDF files to MOL2 formats considering the physiological pH of 7.4).*

*The RMS gradient convergence criterion of 1 × 10⁻⁷ is a very common and strict threshold used computation chemistry and physics for geometry optimizations. This update is now reflected in the Methods section (lines 122-123).*

**Comment**

5. Indicate the file formats generated (e.g., SDF, MOL2, PDB) and detail any further preparation steps used for docking (charge assignment, atom typing, conversion to PDBQT, conformer selection, number of conformers retained).

**Response**

*Thank you for your suggestion. The Structure preparation and file formats are now detailed in the updated manuscript around lines 114 to 160.*

**Comment**

6. For reproducibility, please provide the optimized 3D structures (e.g., as an SDF/MOL2/PDB supplementary file) or a link to a repository containing these geometries.

**Response**

*Thank you for the recommendation. The optimized structures can be accessed via <https://doi.org/10.5281/zenodo.17804558>. This has been included in the manuscript around lines 177-178*.

**Comment**

7. The grid box parameters (center coordinates, box size, and grid spacing) are provided only for the Bcl-2 protein. Since docking was performed against eight target proteins, similar details should be included for all targets to ensure methodological completeness and reproducibility.

**Response**

*Thank you for this important suggestion. For methodological completeness and reproducibility, we have updated the manuscript to include the grid box parameters of for all the eight target proteins and for cleaner and easier to read, we have put this in a table form as a supplemental data.*

**Comment**

8. The manuscript describes post-docking energy minimization using OpenBabel and the Universal Force Field (UFF), which is appropriate for local structural relaxation. However, this step cannot substitute for molecular dynamics (MD) simulation, as energy minimization provides only a static refinement and does not account for time-dependent stability or conformational flexibility of the complex. It is recommended to perform MD simulations to more accurately assess the dynamic stability and binding behavior of the top compound–protein complexes.

**Response**

*We greatly acknowledge your concern, but the decision to use post-docking energy minimization instead of full Molecular Dynamics (MD) simulation, as a reviewer in the initial round of review recommended, was based on computational efficiency and the immediate goal of structural refinement due to resource constraints. Minimization was used because it is significantly faster, making it the only feasible option for high-throughput analysis of the number of docked complexes we had. We used it to efficiently relieve high-energy steric clashes, and structural strains often present in docking poses, thereby ensuring the complex is geometrically valid and resides in the nearest local potential energy minimum. While MD offers complete dynamic and entropic analysis, minimization was a resource-conscious and sufficient method to validate the structural feasibility of the initial binding hypothesis. This limitation has been acknowledged in the revised manuscript around lines 457-462.*

**Comment**

9. Please include the accessible web links for all ADMET and drug-likeness tools for reproducibility and clarity.

**Response**

*We have cross-checked the links and updated them to ensure that they are accurate. This update is located around lines 161 – 170*

**Comment**

10. The authors have discussed only the top-scoring complex in the docking evaluation. It is recommended to include docking results for all other ligand–protein complexes as supplementary data to provide a comprehensive view of binding affinities and interactions across all tested compounds.

**Response**

*We appreciate your observation. However, the docking results for the other compounds have been provided in Table 1.*
