# Supplementary material for: In silico evaluation of garlic-derived organosulfur compounds as multi-target inhibitors of breast cancer biomarkers
Source: PLoS One. 2026 May 6;21(5):e0348369. doi: 10.1371/journal.pone.0348369 (PMC13148666; doi:10.1371/journal.pone.0348369)
Supplement: Attachment — Submitted filename: reviewer 4_response.docx [file pone.0348369.s007.docx]

Reviewer 4 Response

**Comment 1**

The Introduction starts abruptly and lacks sufficient background context. The authors should begin with a brief overview of breast cancer burden, current therapeutic strategies, and existing clinical limitations to provide a logical foundation for the study.

**Response**

Thank you for your comment. In response, we have revised the Introduction to provide clearer background context. Specifically, we now include a brief overview of the global burden of breast cancer (Lines 57-62), followed by discussion of current therapeutic strategies and their limitations, particularly the challenge of multidrug resistance.

**Comment 2**

The statement describing the identification of Z-ajoene and S-allyl-L-cysteine as promising candidates appears to report the study’s findings within the Introduction. Instead, the Introduction should focus on the rationale, knowledge gaps, and study objectives.

**Response**

Thank you for this comment. We agree that the Introduction should focus on the study rationale and knowledge gaps rather than report findings. Accordingly, the Introduction has been revised to remove statements suggesting study outcomes and instead emphasize the background, research gap, and study objectives.

**Comment 3**
The manuscript lacks a clear justification for the selection of the proposed molecular targets in breast cancer. The authors should explicitly explain the biological and clinical relevance of these target proteins, including their roles in breast cancer progression, signaling pathways, and therapeutic relevance. A concise rationale for each target is required.

**Response**

Thank you for this comment. The biological and clinical relevance of the selected molecular targets has been described in the Introduction (Lines 70-79). Specifically, we discuss their roles in key processes associated with breast cancer progression, including apoptosis regulation (Bcl-2 and XIAP), cell cycle control (CDK2 and CDK6), DNA replication and repair (topoisomerases I and II), and tumor angiogenesis (VEGFR2 signaling). In addition, the therapeutic relevance of G-quadruplex DNA structures in oncogene regulation is also highlighted.

**Comment 4**

The authors should confirm whether the selected PDB structures correspond to human proteins. If any structures are derived from non-human homologs, the rationale for their selection and the level of structural or sequence similarity to human proteins should be clearly stated.

**Response**

Thank you for this comment. We confirm that all selected PDB structures correspond to human proteins and are not derived from non-human homologs. To improve clarity, we have revised the Methods section to state this in the revised manuscript (Lines 140-141).

**Comment 5**

Appropriate references for each PDB ID should be provided. The authors are encouraged to cite the original structural studies (X-ray crystallography or cryo-EM publications) rather than only referring to the Protein Data Bank.

**Response**

We appreciate this suggestion. In the revised manuscript, around lines 141 to 145, we have referenced the publications which resolved the structure of the molecular targets in addition to their PDB ID.

**Comment 6**
A comparative analysis with standard FDA-approved breast cancer drugs (e.g., tamoxifen, paclitaxel, doxorubicin, trastuzumab) should be included. Benchmarking docking scores, binding energies, and interaction profiles against standard therapeutics would improve the clinical relevance of the study.

**Response**

Thank you for this suggestion. Comparative discussion with relevant FDA-approved breast cancer therapeutics has been included in the revised manuscript (Lines 398-425). Specifically, we compare the binding interactions and potential mechanisms of the identified compounds with established drugs such as palbociclib, Abemaciclib, etoposide, cediranib, and the G-quadruplex stabilizer quarfloxin. These comparisons highlight similarities in binding modes, interaction with key residues, and potential functional implications, while also acknowledging the relatively lower binding affinities of the garlic-derived compounds and the need for further structural optimization.

**Comment 7**
There is inconsistent use of abbreviations throughout the manuscript. All abbreviations should be defined at first mention and used consistently. Unnecessary or redundant abbreviations should be avoided.

**Response**

Thank you for this comment. We have carefully reviewed the manuscript to ensure that all abbreviations are defined at their first occurrence and used consistently throughout the text. Any redundant or unnecessary abbreviations were also checked and corrected where appropriate to improve clarity.

**Comment 8**

Potential off-target interactions and unintended biological effects of the proposed compounds should be discussed. Incorporating computational off-target prediction would strengthen the safety evaluation.

**Response**

Thank you for this suggestion. In response, we evaluated the potential off-target interactions and unintended biological effects of the proposed compounds through computational ADMET and toxicity predictions. The analysis indicated that P-glycoprotein may be inhibited by compounds cpd2, cpd3, cpd4, and cpd5, while CYP2C9 inhibition was predicted for cpd1 (Z-ajoene). These findings and their potential implications have been discussed in the revised manuscript (Lines 450-458).

**Comment 9**

Molecular dynamics simulations should be performed for at least 200 ns to validate the docking results to ensure stability and convergence of the protein–ligand complexes.

**Response**

Thank you for this suggestion. We agree that molecular dynamics (MD) simulations can provide important information into the stability and convergence of protein-ligand complexes. However, due to logistical and computational constraints, we were unable to perform extended MD simulations during this study. As an alternative, post-docking energy minimization was conducted to refine the docking poses and improve the stability of the predicted complexes. We acknowledge the absence of MD simulations as a limitation of the present study and have explicitly stated this in the manuscript.

**Comment 10**
A comparative discussion with previously published studies is missing. The authors should compare their findings with existing computational and experimental reports on similar targets and compounds, highlighting similarities, differences, and the novelty of the present work. This will help position the study within the current literature and clarify its scientific contribution.

**Response**

Thank you for this great suggestion. We agree that positioning our findings within the context of existing literature strengthens the scientific contribution of the study. Accordingly, we have revised the Discussion section (Lines 426-441) to include comparative analysis with previously published computational and experimental studies involving similar molecular targets and natural compounds. These additions highlight similarities and differences in binding interactions, docking affinities, and reported biological activities, while also emphasizing the novelty and potential therapeutic relevance of the garlic-derived compounds investigated in this study.
